# Who is the culprit: Is pest infestation responsible for crop yield losses close to semi‐natural habitats?

**DOI:** 10.1002/ece3.8046

**Published:** 2021-08-24

**Authors:** Larissa Raatz, Karin Pirhofer Walzl, Marina E. H. Müller, Christoph Scherber, Jasmin Joshi

**Affiliations:** ^1^ Institute of Biochemistry and Biology Universität Potsdam Universität Potsdam Potsdam Germany; ^2^ Leibniz Centre for Agricultural Landscape Research (ZALF) e.V Müncheberg Germany; ^3^ Institute at Brown for Environment and Society Brown University Providence RI USA; ^4^ Berlin‐Brandenburg Institute of Advanced Biodiversity Research (BBIB) Berlin Germany; ^5^ Institute of Biology Freie Universität Berlin Berlin Germany; ^6^ Zoological Research Museum Alexander Koenig (ZFMK) Centre for Biodiversity Monitoring Bonn Germany; ^7^ Institute for Landscape and Open Space Eastern Switzerland University of Applied Sciences Jona‐Rapperswil Switzerland

**Keywords:** arable weeds, cereal leaf beetle, fungal pathogens, herbivory, structural equation model, wheat

## Abstract

Semi‐natural habitats (SNHs) are becoming increasingly scarce in modern agricultural landscapes. This may reduce natural ecosystem services such as pest control with its putatively positive effect on crop production. In agreement with other studies, we recently reported wheat yield reductions at field borders which were linked to the type of SNH and the distance to the border. In this experimental landscape‐wide study, we asked whether these yield losses have a biotic origin while analyzing fungal seed and fungal leaf pathogens, herbivory of cereal leaf beetles, and weed cover as hypothesized mediators between SNHs and yield. We established experimental winter wheat plots of a single variety within conventionally managed wheat fields at fixed distances either to a hedgerow or to an in‐field kettle hole. For each plot, we recorded the fungal infection rate on seeds, fungal infection and herbivory rates on leaves, and weed cover. Using several generalized linear mixed‐effects models as well as a structural equation model, we tested the effects of SNHs at a field scale (SNH type and distance to SNH) and at a landscape scale (percentage and diversity of SNHs within a 1000‐m radius). In the dry year of 2016, we detected one putative biotic culprit: Weed cover was negatively associated with yield values at a 1‐m and 5‐m distance from the field border with a SNH. None of the fungal and insect pests, however, significantly affected yield, neither solely nor depending on type of or distance to a SNH. However, the pest groups themselves responded differently to SNH at the field scale and at the landscape scale. Our findings highlight that crop losses at field borders may be caused by biotic culprits; however, their negative impact seems weak and is putatively reduced by conventional farming practices.

## Graphical abstract

Understanding how semi‐natural habitats influence provisioning ecosystem services of agricultural fields should be a key argument in debates about economic profits versus biodiversity conservation in agricultural landscapes. Therefore, we studied the effect of pest infestation as a biotic culprit to yield losses close to hedgerows and kettle holes using a structural equation model. We found arable weed cover being associated with yield losses along the transects, however, only in the proximate distances to the field border.

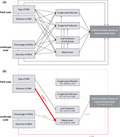

## 1 INTRODUCTION

Intensification of agriculture has led to a depletion of semi‐natural habitats (SNHs) in agricultural landscapes and to associated losses of biodiversity (Foley, 2005; Sala et al., 2000; Tilman et al., 2002). However, SNHs have been shown to provide important biodiversity‐mediated ecosystem services (Tscharntke et al., 2005) such as pollination (Bianchi et al., 2006; Garibaldi et al., 2011) and pest control (Chaplin‐Kramer et al., 2011; Veres et al., 2013). Especially, landscape complexity, in terms of amount and diversity of SNHs, was reported to be of major importance for beneficial species (Chaplin‐Krameret al., 2011; Rusch et al., 2013; Tscharntke et al., 2007), whereas pest populations often showed inconsistent responses to higher landscape complexity (Karp et al., 2018; Papaïx et al., 2011). Several studies observed a decrease in pest densities with increasing landscape complexity (Chaplin‐Kramer & Kremen, 2012) either due to an effective pest control mediated through SNHs (Rusch et al., 2016) or due to a reduced amount of cropped habitat (Dominik et al., 2018). However, SNHs can also act in favor of pest populations as they provide alternative food resources or refugia against agricultural disturbances (Tscharntke et al., 2016). Thus, SNHs provide habitats not only for natural enemies or pollinators but also for pests that subsequently may spillover from SNHs into agricultural fields (Blitzer et al., 2012). Perez‐Alvarez et al. (2018) observed that even the presence of a single SNH type, here meadows, can produce mixed pest responses reducing one pest species, but augmenting another that spilled‐over from the SNH into agricultural fields. Hence, understanding the role of SNHs on pest population dynamics and corresponding crop damage is a prerequisite for developing ecologically sustainable crop protection strategies in order to reduce intensive use of agrochemical inputs (Skellern et al., 2017).

Still, it has remained largely unexplored which particular types of SNHs and their distribution in the landscape contribute to an optimal provision of ecosystem services and potentially also to increasing crop yield. According to Holland et al. (2017), only few studies have made the attempt to evaluate the effect of SNHs on yields representing the most valued service for farmers in agricultural landscapes.

A global synthesis has recently revealed that landscape complexity can increase yields mediated through predator and pollinator richness (Dainese et al., 2019). Further, Liere et al. (2015) found that with increasing habitat diversity cascading effects from high predator abundances led to lower pest densities, less plant damage, and therewith slightly increased yields. However, if high landscape complexity favors species from higher trophic levels, trophic cascades may be indirectly beneficial to herbivores, again resulting in a yield decrease (Martin et al., 2013). These contrasting responses of pest populations and their consequences for crop yield have also been frequently studied at a field scale, where especially the effects of hedgerows and grass strips have been in focus (Holland et al., 2016; Van Vooren et al., 2017). Although hedgerows have been shown to enhance parasitism and pollination (Dainese et al., 2017), the direct impacts of tall vegetation structures at field borders often lead to lower yields due to shading and competition for nutrients and water (Kort, 1988; Kowalchuk & Jong, 1995). This emphasizes a trade‐off between crop production and regulating ecosystem services next to woody habitats (Van Vooren et al., 2018). Grass strips generally have been reported to increase pest control, with lower pest densities having either no consequences (Albrecht et al., 2020) or positive effects on yield (Gurr et al., 2016; Tschumi et al., 2015). In a recent study, we demonstrated that the effect of SNHs on yield varies between SNH types (Raatz et al., 2019). In proximity to woody structures, such as forest borders and hedgerows (which were neither trimmed nor surrounded by an extensively managed strip of grassland), yield losses were higher compared with field‐to‐field borders, whereas in the vicinity of kettle holes, yield losses were negligible.

In the present study, we aim to shed light on the biotic culprits of yield losses close to SNHs by focusing on four different wheat‐specific pests (Oerke, 2006) potentially associated with SNHs and entailing ecosystem disservices by reducing crop production. We chose fungal seed pathogens, fungal leaf pathogens, cereal leaf beetle larvae (*Oulema* spp., hereafter CLB), and arable weeds. The taxa studied represent the major pest groups of winter wheat in the temperate zone with pathogens accounting for 10% of global wheat yield losses, followed by animal pests and weeds (each 8%) in the presence of crop protection practices (Oerke, 2006). Adapted to the rapid ecosystem function assessment method (Meyer et al., 2015), we measured fungal seed and leaf infection rates, herbivory rates, and weed cover to relate these pest groups to yield.

While the effect of SNHs for weeds is intensively studied (Fried et al., 2009), less is known about the role of SNHs for fungal pathogens and CLB larvae (Holland et al., 2017, but see Tschumi et al., 2015). Therefore, we additionally assessed the effect of SNHs also on those wheat pests. To investigate whether the response of pest rates to SNH type and the distance to SNH is altered by increasing landscape complexity, we extended our scope to the landscape scale by accounting for the percentage and diversity of SNHs within a radius of 1,000 m.

Our approach is based on experimental plots where a single wheat variety is sown at several in‐field points in conventionally managed winter wheat fields. This allows to measure seed biomass and pest rates in a highly standardized way across different agricultural fields.

To find the culprit of yield losses associated with SNHs, we asked (i) whether these crop losses have a biotic origin. More specifically, we were interested which of the selected pests’ rates (fungal seed and leaf infection, herbivory of CLB, and weed cover) influenced wheat yield and (ii) if their effects were related to the type and distance of a SNH at the local scale and the percentage and diversity at a landscape level.

## 2 METHODS

### 2.1 Study area

We conducted our study from September 2015 to July 2016 at the ZALF research platform “AgroScapeLab Quillow” (250 km^2^, Uckermark, Brandenburg, Germany). This area is characterized by a subcontinental climate with 8.7℃ mean annual temperature and a low mean annual precipitation of 475 mm/year (ZALF field station, Dedelow). The landscape is dominated by agricultural fields and grasslands (62%) interspersed with forests (24%), water bodies (5%), and small settlements (5%). Frequent semi‐natural habitats (SNHs) in the catchment are tall hedgerows along fields as well as kettle holes—small water bodies (<1 ha, Kalettka & Rudat, 2006)—within fields as remnants of the last ice age. More than one third of agricultural fields in the area are cultivated with winter wheat with an average field size of 46 ha (Amt für Statistik Berlin‐Brandenburg, 2014) that yields on average 7.4 t/ha (Amt für Statistik Berlin‐Brandenburg, 2017, 2018).

### 2.2 Study design

We selected twelve winter wheat fields based on the biotope map of Brandenburg 2009 (Landesamt für Umwelt, Brandenburg, Germany) and the regional land‐use data of the “AgroScapeLab Quillow” from 2015 and 2016 in ArcGIS 10.4.1 (ESRI, Redlands, California, USA). Selected fields had to meet the following criteria to make them comparable: (i) winter wheat as the main crop in 2016, (ii) oil‐seed rape as the winter wheat precrop in 2015, and (iii) a field size ranging from 20 to 75 ha. In each field, one transect was established from an adjacent SNH being either a hedgerow (*N* = 6) or a kettle hole (*N* = 6) into the winter wheat field with four distances at a modified logarithmic scale (1 m, 5 m, 20 m, and 50 m; see Raatz et al., 2019 for details). The selected hedgerows had a total of 22 woody species, while *Sambucus nigra*, *Prunus spinosa,* and *Rosa* spec. were most frequently represented. They consisted mainly of a loose to dense shrub layer ranging from 4 to 7 m being often interspersed by fruit trees or higher tree species such as *Acer* spec. or *Salix alba* reaching up to 12 m.

### 2.3 Experimental plots

At each wheat field (*N* = 12), we established one experimental plot at each of the four distances along the transect within a 1‐m^2^ area (yielding *N* = 48) by sowing 300 seeds of the winter wheat variety “Julius,” a frequently used variety for the study area, with a manual sowing machine into a 1‐m² plot composed of 6 rows (inter‐row distance = 12 cm with 300 seeds/m^2^). All experimental plots were managed identically as the crop wheat plants on the field by the farmer, except of a delayed sowing date of four weeks. Winter wheat in the study area is generally treated with three to four fertilizer applications per year (total: 180–220 kg N/ha). Pesticides are applied according to infestation rates, whereof generally in autumn a herbicide is dispersed against annual weeds and in spring up to three fungicide treatments against root diseases, *Septoria*, brown rust or *Fusarium*. In general, the only insecticide employed by farmers is against aphids depending on animal abundance.

Experimental plots were harvested aboveground at seed maturation (growth stage 87–89), threshed with a laboratory threshing machine, and dried at 65℃ for 48 hr to constant dry weight. Seed biomass was weighed and converted to grain yield in t/ha. To validate our experimental approach, we compared the yield of our sown wheat plants (var. Julius) with the yield of winter wheat sown by the farmer (var. Akteur, Discus, Julius, Patras, Reform or Rumor; hereafter field wheat) harvested in the same design (for further details, see dataset of 2016 in Raatz et al., 2019).

### 2.4 Selected pest groups of wheat

To assess potential yield losses at increasing distances to hedgerows and kettle holes (SNH types), we measured rates of fungal pathogens (on seeds and leaves) as well as one animal pest (CLB larvae) of winter wheat directly on the plants of our experimental plots and arable weeds, as main competitors of crop plants for nutrients, light, and space, next to the experimental plants.

#### 2.4.1 Fungal seed infection

Ten wheat ears were collected per experimental plot prior to harvest at a wheat growth stage of 83–85. Ten seeds of the wheat ears per plot were randomly selected, the outer husks were removed, and the naked seeds were incubated on potato dextrose agar (PDA) for three days at 25℃ in darkness followed by three days with a 12‐hr/12‐hr black light (emission 310–360 nm)/darkness cycle. A total fungal infection rate was calculated by counting the fungal colonies (colony‐forming units, CFU) and extrapolated to number of CFUs per 100 seeds. Additionally, the phytopathogenic fungi of the genera *Alternaria* and *Fusarium* were taxonomically determined and counted as genus‐specific CFUs per 100 seeds.

#### 2.4.2 Fungal leaf infection

Fungal leaf pathogens (mainly brown and yellow rust, powdery mildew, and *Septoria* spp.) were visually inspected on three flag leaves per row (*N* = 18 leaves per plot) at a wheat growth stage of 75 – 77. We only studied flag leaves as they contribute up to 60% to grain yield, whereas leaves below the flag only modestly contribute to grain yield (Thorne, 1966). Here as well, we recorded an unspecific fungal infection rate (independently of fungal group) as the proportion of infected flag leaves to the total number of investigated flag leaves per experimental plot (*N* = 48).

#### 2.4.3 Herbivory of CLB

Herbivory was recorded by visually detecting the characteristic feeding patterns of the CLB larvae on three flag leaves per row (*N* = 18 leaves per plot) at a wheat growth stage of 75–77. CLB larvae skeletonize the leaves by feeding on all leaf tissue except the lower epidermis (Gallun et al., 1967), which reduces the photosynthetic capacity of the plant resulting in a decline of the number of grains per spike and the thousand‐grain weight. A damage threshold for wheat where feeding damage leads into measurable yield losses is often indicated at 10% loss of flag leaf area corresponding to about 10% yield loss (Hoffmann & Schmutterer, 1999; Kirch, 2006). We therefore quantified herbivory rate as the proportion of leaves damaged by more than 10 percent, relative to the total number of investigated leaves per experimental plot (*N* = 48), using a scoring scale from 1 (no damage) to 9 (very severe damage) adapted from Moll et al. (2000) (Table S1). Within the herbivory dataset, we had two missing data points that originated from a too low number of flag leaves for a representative recording of herbivory at two experimental plots. We replaced these two data points by the median of all other herbivory rates.

#### 2.4.4 Weed cover

We recorded total cover of weeds using a Braun‐Blanquet (1951) scale in six 1‐m^2^ vegetation quadrats close to each experimental plot at a wheat growth stage of 37–45 with three quadrats to the left and three quadrats to the right of the plot, parallel to the field border. Single scores were converted to percentage values (Table S2) and averaged per experimental plot (*N* = 48).

### 2.5 Landscape complexity

We were interested whether landscape complexity altered putative effects on yield of the four selected pests at the field scale. Therefore, we analyzed the effect of percentage and diversity of SNHs within a 1000‐m radius around each experimental plot on fungal seed and leaf infection, herbivory of CLB larvae, and weed cover. We identified water bodies (including kettle holes), ruderal areas, fens, grasslands, hedgerows, and forests to represent SNHs in our study area and calculated the percentage as well as the Shannon diversity index (SDI) of these six biotope classes with Fragstats 4.2 (McGarigal & Marks, 1995) using the eight‐cell‐neighborhood rule in a grid of 1 m × 1 m within a 1,000‐m radius around each experimental plot (*N* = 48). Percentage of SNHs ranged from 4.7% to 27.4% and SDI of SNHs from 0.74 to 1.58. These compositional landscape measures, percentage and diversity of SNHs, were not significantly correlated to each other (*r* (46) = 0.27, *p* > 0.05).

### 2.6 Statistical analyses

#### 2.6.1 Single mixed‐effects models

To unravel the biotic culprit of yield losses due to SNHs, we first analyzed the effects of fungal seed and fungal leaf infection rates, herbivory rates, and weed cover on winter wheat yield in our experimental plots, as well as the local and landscape effects of SNHs on the selected pests performing several single linear and generalized linear mixed‐effects models in the packages “nlme” (Pinheiro et al., 2018) and “lme4” (Bates et al., 2015) in R version 4.0.3. (R Core Team, 2020).

As a prerequisite and to verify our experimental approach, we first analyzed the yields of our experimental plots and the field wheat of 2016 in a joint mixed model (model 1; Table 1; *N* = 95) by entering: *yield ~yield type (experimental plots* vs. *agricultural field)* * *distance to a SNH* * *SNH type (hedgerow* vs. *kettle hole), random* *= 1|field*. From the field wheat dataset, we excluded one outlier that would have strongly affected the results (*N* = 47). As the visual inspection of residual plots with either yield type as response variable did not reveal any obvious deviations from homoscedasticity or normality, we performed a linear mixed‐effects model. “Distance to a SNH” was log‐transformed for a better fit of the model. Random effects of field identity were included to account for field variability. Log‐transformation of distance and field identity as random effect was maintained throughout all further models.

**TABLE 1:** Type II analysis of variance for the linear mixed‐effects model (model 1) on winter wheat yield as a function of yield type (experimental plots vs. field wheat), distance to a SNH (1 m, 5 m, 20 m, 50 m), SNH type (hedgerow, kettle hole), and all their interaction terms; bold font: significant (*p* < 0.05)

	Chisq	*df*	*p*‐Value
Yield type	**48.69**	**1**	**<0.001**
Distance to a SNH	**38.04**	**1**	**<0.001**
SNH type	2.30	1	0.130
Yield type × distance	**4.56**	**1**	**0.033**
Yield type × SNH type	0.01	1	0.922
Distance × SNH type	**8.10**	**1**	**0.004**
Yield type × distance × SNH type	0.02	1	0.883
Random effect (1| field)	*SD* = 0.62		

Model 1: [yield ~yield type * distance to a SNH * SNH type, random = 1|field] (*N* = 95).

The table is the result from a likelihood ratio chi‐square test with individual model terms taking up only 1 degree of freedom (*df*).

For direct effects of the selected pests on wheat yield of the experimental plots (*N* = 48), we performed a linear mixed‐effects model (model 2a; Table 2): *yield ~ (distance to a SNH + SNH type)* * *(fungal seed infection* *+ fungal leaf infection + herbivory + weed cover) + distance to a SNH:SNH type, random = 1|field*. As reported in Raatz et al. (2019), yield losses were only detectable within the two most proximate distances to a SNH along our transects. Hence, we repeated our analysis using only yield values of the experimental plots at 1‐m and 5‐m distance to an adjacent SNH (*N* = 24) omitting the fixed effect “Distance to a SNH” and its interaction terms from the model (model 2b): *yield ~SNH type* * *(fungal seed infection* *+ fungal leaf infection + herbivory + weed cover), random = 1|field*.

**TABLE 2:** Type II analysis of variance for linear mixed‐effects models (2a and 2b) examining relationships between local factors of SNHs (distance to SNH and SNH type) and fungal seed and fungal leaf infection, herbivory of CLB larvae, and weed cover on winter wheat yield of experimental plots; bold font: significant (*p* < 0.05)

	With all distances^a^			Only with proximate distances^b^		
	Chisq	*df*	*p*‐Value	Chisq	*df*	*p*‐Value
Distance to SNH	3.93	1	**0.048**	–	–	–
SNH type	0.57	1	0.450	1.71	1	0.190
Distance × SNH type	1.47	1	0.226	–	–	–
Fungal seed infection	1.67	1	0.196	1.02	1	0.313
Fungal leaf infection	0.00	1	0.954	0.01	1	0.916
Herbivory of CLB	0.05	1	0.824	0.00	1	0.962
Weed cover	0.91	1	0.341	3.49	1	0.062
Distance × Seed infection	0.02	1	0.891	–	–	–
Distance × Leaf infection	0.17	1	0.683	–	–	–
Distance × Herbivory	0.75	1	0.385	–	–	–
Distance × Weed cover	0.11	1	0.739	–	–	–
SNH type × Seed infection	0.87	1	0.351	0.43	1	0.512
SNH type × Leaf infection	0.90	1	0.343	0.28	1	0.596
SNH type × Herbivory	0.89	1	0.346	0.01	1	0.907
SNH type × Weeds	0.42	1	0.518	0.21	1	0.649
Random effect (1| field)	*SD* = 0.93			*SD* = 0.55		

^a^ Model 2a including exp. plots at all four distances (1 m, 5 m, 20 m, 50 m): [yield ~ (distance to a SNH + SNH type) * (fungal seed infection + fungal leaf infection + herbivory + weed cover) + distance to a SNH × SNH type, random = 1|field] (*N* = 48).

^b^ Model 2b including only exp. plots at the two proximate distances (1 m and 5 m): [yield ~SNH type * (fungal seed infection +fungal leaf infection +herbivory + weed cover), random = 1|field] (*N* = 24).

The table is the result from a likelihood ratio chi‐square test with individual model terms taking up only 1 degree of freedom (*df*).

Furthermore, each selected pest group was analyzed separately concerning local and landscape factors of SNHs on their rates. Here, we entered the following fixed effects in four single models (models 3a–3d; Table 3): *pest ~distance to a SNH* * *SNH type* + Percentage *of SNH* * (*distance to a SNH* + SNH *type)* + *Diversity of SNH* * (*distance to a SNH* + SNH *type), random* *= 1|field*. For fungal leaf infection rate and herbivory rate, we used binomial distributions in generalized linear mixed‐effects models. For fungal seed infection rate and weed cover, we used linear mixed‐effects models even though weed cover had to be log‐transformed to obtain normality.

**TABLE 3:** Type II analysis of variance for (generalized) linear mixed‐effects models (3a – 3d) examining relationships between local (distance to SNH and SNH type) and landscape factors (percentage and diversity) of SNHs on fungal seed and fungal leaf infection, herbivory of CLB larvae, and weed cover; bold font: significant (*p* < 0.05)

	Fungal seed infection^a^			Fungal leaf infection^a^		
	Chisq	*df*	*p*‐Value	Chisq	*df*	*p*‐Value
Distance to a SNH	0.00	1	0.995	2.99	1	0.084
SNH type	0.02	1	0.896	0.46	1	0.497
Percentage of SNHs [%SNH]	1.53	1	0.216	0.23	1	0.633
Diversity of SNHs [SDI]	0.73	1	0.394	0.02	1	0.882
Distance × SNH type	0.63	1	0.429	16.50	1	**<0.001**
Distance × %SNH	0.15	1	0.702	15.85	1	**<0.001**
SNH type × %SNH	3.04	1	0.081	0.31	1	0.579
Distance × SDI	0.38	1	0.537	0.02	1	0.895
SNH type × SDI	0.23	1	0.629	3.61	1	0.057
Random effect (1| field)	*SD* = 51.74			*SD* = 2.27		

	Herbivory of CLB^a^			Weed cover^a^, ^b^		
	Chisq	df	*p*‐Value	Chisq	df	*p*‐Value
Distance to a SNH	1.56	1	0.212	31.80	1	**<0.001**
SNH type	5.42	1	**0.020**	0.05	1	0.827
Percentage of SNHs [%SNH]	0.02	1	0.876	5.04	1	**0.025**
Diversity of SNHs [SDI]	0.00	1	0.976	0.53	1	0.467
Distance × SNH type	0.62	1	0.431	0.98	1	0.323
Distance × %SNH	0.71	1	0.399	0.08	1	0.773
SNH type × %SNH	0.21	1	0.650	1.62	1	0.203
Distance × SDI	0.08	1	0.777	1.06	1	0.304
SNH type × SDI	5.58	1	**0.018**	1.92	1	0.166
Random effect (1| field)	*SD* = 0.59			*SD* = 0.65		

^a^ Models 3a–3d: [pest ~ distance to a SNH * SNH type + percentage of SNH * (distance to a SNH + SNH type) + diversity of SNH * (distance to a SNH + SNH type), random = 1|field] (*N* = 48 for each pest group).

^b^ Weed cover is log‐transformed.

The table is the result from a likelihood ratio chi‐square test with individual model terms taking up only 1 degree of freedom (df).

#### 2.6.2 Structural equation modeling using a piecewise approach (SEM)

In a second step, we combined several mixed‐effects models used a structural equation model (*SEM*) with the package “piecewiseSEM” (Lefcheck, 2016) to investigate the indirect effects of SNHs on yields of our experimental plots mediated by fungal seed and fungal leaf infection rates, herbivory rates, and weed cover. Piecewise structural equation models allow to investigate networks of hypotheses that would be difficult to assess using individual mixed models only. To comply with our hierarchical study design and relatively low sample size, we chose to conduct a “one model only” *SEM* following Grace et al. (2015), rather than testing multiple alternative SEMs. For the meta‐model, we specified a total of five linear mixed‐effects models, where each endogenous variable (yield, fungal seed infection, fungal leaf infection, herbivory, and weed cover) was related to the four exogenous local and landscape factors of SNHs (distance to a SNH, SNH type, percentage of SNHs, and diversity of SNHs), always including field identity as a random effect (Table 4). A direct path was added from each of the four pest groups to yield. Fungal leaf infection and herbivory were logit‐transformed and weed cover log‐transformed to achieve a normal distribution of residuals and a better model fit. Beyond that we interlinked the four pests to each other in order to capture a more realistic picture: We hypothesized that higher weed cover benefits fungal infection and herbivory because arable weeds may serve as alternative sources for seed and leaf fungi within the field (Wisler & Norris, 2005) and as alternative host plants for CLB (Glogoza, 2002) to recolonize the wheat plant. Furthermore, we assumed that fungal leaf infection rates would be affected positively by herbivory rates because fungi may enter more easily if the plant tissue is destroyed (e.g., Munkvold, 2003).

**TABLE 4:** Summary table of linear mixed‐effects models in final piecewise *SEM* (C_30_ = 15.33; *p* = 0.988) examining relationships between local (type of SNH and distance to SNH) and landscape factors (percentage and diversity within 1,000 m radius around the plots) of SNHs and winter wheat yield of experimental plots, fungal seed and fungal leaf infection, herbivory of CLB larvae, and weed cover; bold font: significant (*p* < 0.05), gray font: nonsignificant (*p* > 0.1); each variable *N* = 48

Response	Predictor	Estimate	Std. Estimate	Std. Error	*df*	*p*‐Value
Yield	Distance to a SNH	0.189	0.207	0.126	31	0.143
	%SNH	−0.060	−0.398	0.031	31	0.060
	SDI	−1.553	−0.270	1.017	31	0.137
	Leaf infection	0.131	0.156	0.138	31	0.351
	Weed cover	−0.316	−0.293	0.180	31	0.089
		*R* ^2^_marginal_ = 0.32		*R* ^2^_conditional_ = 0.51		
Seed infection	Distance to a SNH	−4.679	−0.108	4.808	34	0.337
	Weed cover	−9.950	−0.194	7.246	34	0.179
		*R* ^2^_marginal_ = 0.03		*R* ^2^_conditional_ = 0.70		
Leaf infection	Distance to a SNH	−0.110	−0.101	0.103	32	0.298
	%SNH	0.059	0.326	0.050	32	0.247
	Herbivory of CLB	−0.425	−0.188	0.268	32	0.123
	Weed cover	−0.211	−0.164	0.163	32	0.203
		*R* ^2^_marginal_ = 0.17		*R* ^2^_conditional_ = 0.82		
Herbivory	Weed cover	−0.108	−0.189	0.067	35	0.114
	Type of SNH	–	–	–	1	0.083
	hedgerow	−2.583	–	0.234	11	–
	kettle hole	−2.008	–	0.234	10	–
		*R* ^2^_marginal_ = 0.17		*R* ^2^_conditional_ = 0.64		
Weed cover	Distance to a SNH	**−0.439**	**−0.522**	**0.079**	**34**	**<0.001**
	%SNH	−0.048	−0.345	0.024	34	0.051
		*R* ^2^_marginal_ = 0.38		*R* ^2^_conditional_ = 0.60		

The *SEM* was simplified by successively removing paths with the highest *p*‐values until further removals did not further decrease the overall model fit. Goodness of fit was assessed based on Shipley's test of directed separation that combines the *p*‐values of all independent claims in Fisher's C (Shipley, 2009). At last, we manually calculated direct, indirect, and total effects of the remaining local and landscape factors of SNHs on yield mediated through the selected drivers based on Finney (1972).

## 3 RESULTS

### 3.1 Winter wheat yields

**FIGURE 1:**
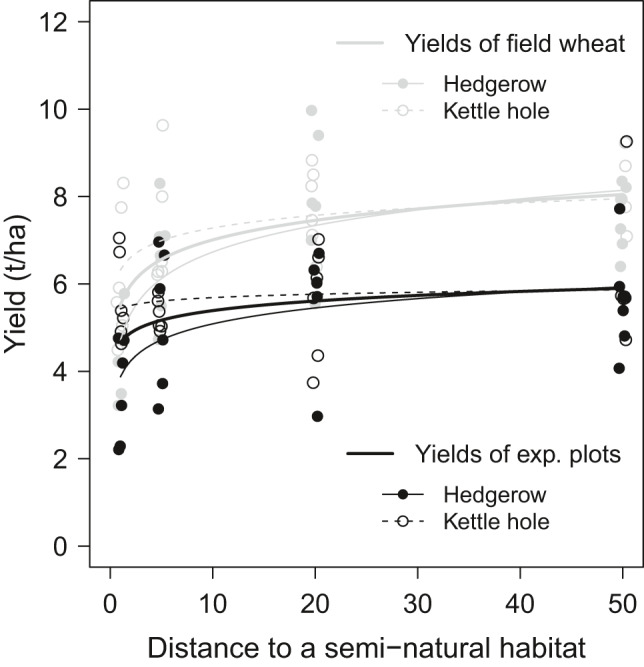
Effect of distance to a semi‐natural habitat (SNH) per SNH type (hedgerow: solid line and kettle hole: dashed line) on winter wheat yield measured as seed biomass in t/ha of experimental plots (*N* = 48; black) and field wheat (*N* = 47; gray). Curves represent fitted values according to the linear mixed‐effects model 1 (Table 1)

Overall, winter wheat plants of experimental plots (that were sown approximately four weeks later than the agricultural crops) yielded on average less (5.3 ± 1.4 t/ha) than winter wheat plants sown by the farmer (6.9 ± 1.6 t/ha; χ^2^ (1) = 48.7, *p* < 0.001; Table 1), which may be due to the delayed sowing time. Concerning the effects of SNHs (distance and type), yields measured at experimental plots and yields measured directly from the field showed similar responses: i. Yields increased with distance to an adjacent SNH (χ^2^ (1) = 38.0, *p* < 0.001; Figure 1; Table 1) and ii. yields were lower close to hedgerows compared with those close to kettle holes increasing more steeply to mid‐field yields with increasing distance to a hedgerow compared with a kettle hole (χ^2^ (1) = 8.1, *p* < 0.01, see also Raatz et al., 2019). Even though the latter effect was independent of yield type, field yields increased more steeply from the field border toward the field center compared with yields of experimental plots (χ^2^ (1) = 4.6, *p* < 0.05). This may point out that the variety we chose for our experimental plots was less affected by SNHs compared to other varieties. Still, in the experimental plots the major trends are reflected verifying our experimental approach and allowing us to control for variety when looking at the effect of the selected pests emanating from these SNHs.

**FIGURE 2:**
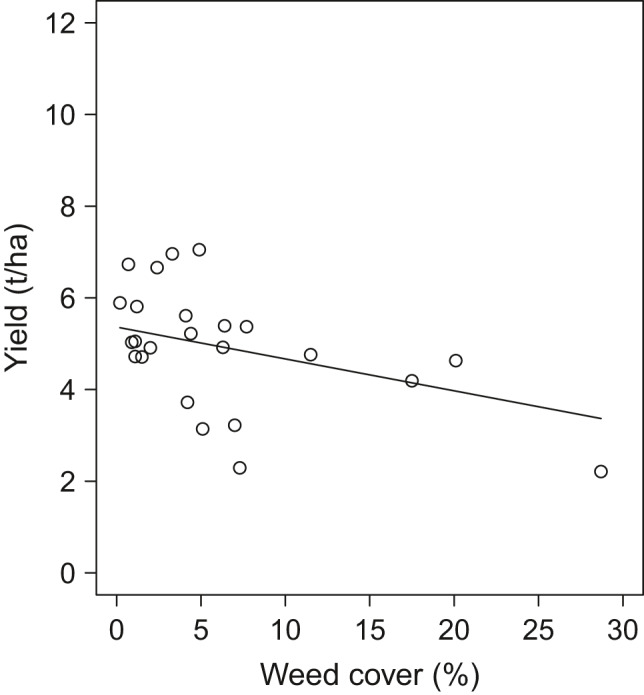
Weed cover (%) at each experimental plot correlated with winter wheat yield measured as seed biomass in t/ha of the experimental plots at 1‐m and 5‐m distances to a SNH (*N* = 24). Curve represents fitted values according to a linear mixed‐effects model 2b (Table 2)

Analyzing the impact of fungal infection rates, herbivory rate, and weed cover on yield of experimental plots, the first model including yield at all distances (Table 2) revealed that none of the pests affected yield significantly, neither solely, nor depending on the distance to a SNH nor on the SNH type (Figure S1). However, when only analyzing yield close to field borders (at 1‐m and 5‐m distances; Table 2), arable weeds reduced wheat yield up to 49% (χ^2^ (1) = 3.5, *p* < 0.1; Figure 2). Even though this negative effect was only marginal, it was independent of SNH type at the field border (χ^2^ (1) = 0.2, *p* > 0.05; Table 2).

#### 3.1.1 Effects of SNHs on pests

Fungal infection on wheat seeds of experimental plots ranged from 120 to 400 colony‐forming units (CFUs) per 100 wheat seeds with a median of 290 CFUs. Most colonies could be attributed to *Alternaria* species (mean: 46% ± 12%) whereas *Fusarium* species were little represented (mean: 2% ± 6%). Fungal infection rates on the sampled wheat seeds were neither affected by distance to a SNH (χ^2^ (1) = 0.0, *p* > 0.05; Table 3) nor affected by SNH type (χ^2^ (1) = 0.0, *p* > 0.05), by any metrics of landscape composition (%SNH: χ^2^ (1) = 1.5, *p* > 0.05; SDI: χ^2^ (1) = 0.7, *p* > 0.05), and nor by the interaction between field and landscape scale.

**FIGURE 3:**
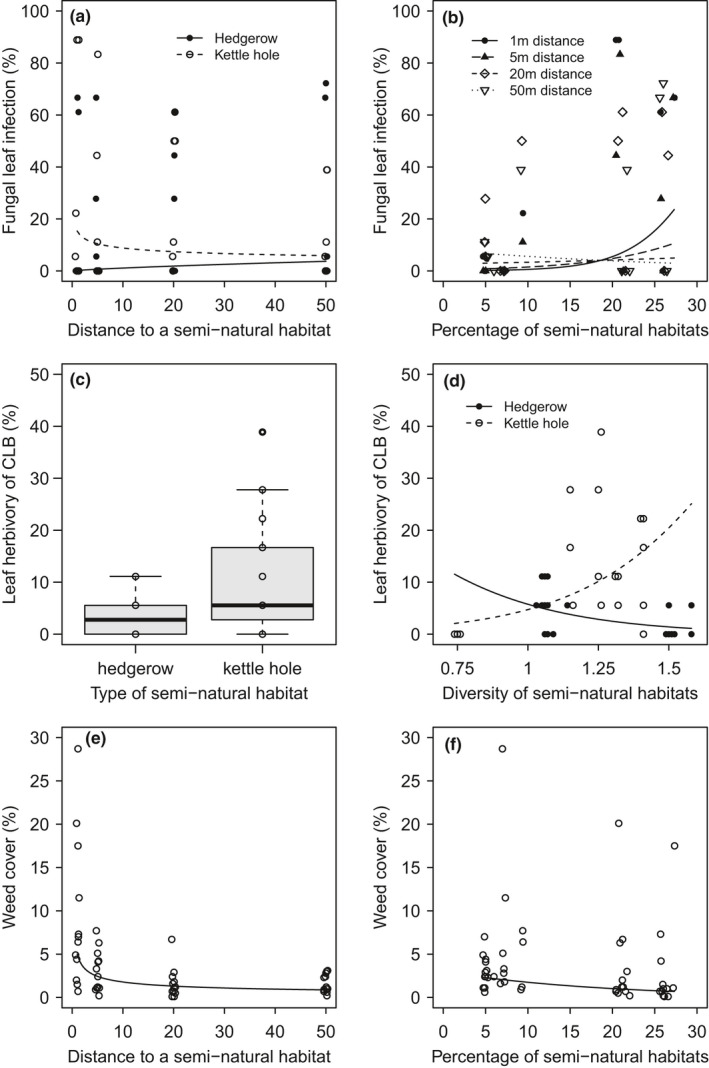
Local (left) and landscape (right) factors of semi‐natural habitats (SNHs) on a + b the percentage of fungal leaf infection, c + d the percentage of herbivory (>10% per leaf) caused by CLB larvae, and e + f weed cover (each pest group with *N* = 48). Percentage of SNHs and Shannon diversity of SNHs were calculated within a radius of 1,000 m around each experimental plot. Curves represent fitted values according to generalized linear mixed‐effects models (3b + 3c) with binomial distribution for fungal leaf infection rate and herbivory of CLB and according to linear mixed‐effects model (3d) for weed cover (log‐transformed). All drawn relationships are significant (*p* < 0.05). Points of subfigures a and e (with distance as explanatory variable) jitter by 0.5

Fungal infection on winter wheat flag leaves ranged from 0% to 88.9% with a median of 5.6%. Close to kettle holes, infection rates decreased from field border to field center by 62%, whereas close to hedgerows rates remained nearly unchanged (χ^2^ (1) = 16.5, *p* < 0.001; Figure 3a). With increasing percentage of SNHs at the landscape scale, fungal infection increased depending on distance to a SNH (χ^2^ (1) = 15.9, *p* < 0.001; Figure 3b): At low percentages of SNHs, infection rates were similarly low at all distances whereas the more SNHs were present in a radius of 1,000 m the more leaves were infected close to the SNH (22% infection rate at 27% SNH) compared with leaves of wheat plants further in the wheat field (5% infection rate at 27% SNH).

Herbivory rates greater than 10% of the leaf surface caused by CLB larvae on flag leaves ranged from 0% to 38.9% with a median of 5.6%. More flag leaves were damaged at kettle holes (10.4%) compared with leaves at hedgerows (2.8%; χ^2^ (1) = 5.4, *p* < 0.05; Figure 3c), and with increasing diversity of SNHs in a 1000‐m radius around experimental plots, the percentage of damaged leaves greater than 10% increased on plots at kettle holes whereas at hedgerows herbivory rates decreased (χ^2^ (1) = 5.6, *p* < 0.05; Figure 3d).

Percentage weed cover ranged from 0.1% to 28.7% with a median of 2.2%. Weed cover was affected by distance to a SNH (χ^2^ (1) = 31.8, *p* < 0.001; Figure 3e) and by percentage of SNHs at the landscape scale (χ^2^ (1) = 5.0, *p* < 0.05; Figure 3f) whereby both, increasing distance but also increasing percentage of SNHs, decreased weed cover by 83% and 69%, respectively.

#### 3.1.2 Indirect effects of SNHs on wheat yield

**FIGURE 4:**
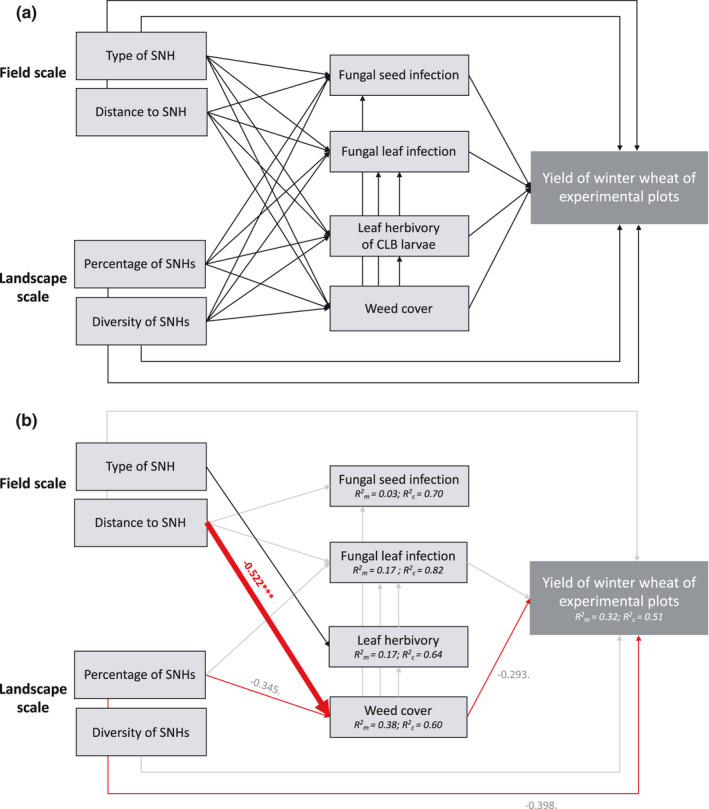
a. Meta‐model and b. final *SEM* (C_30_ = 15.33; *p* = 0.988) analyzing the relationship between semi‐natural habitats at field scale (type of SNH and distance to SNH) and landscape scale (percentage and diversity of SNHs within 1,000 m radius around the plots) and winter wheat yield of experimental plots, fungal seed infection, fungal leaf infection, herbivory of CLB larvae, and weed cover. Black arrows indicate positive and red arrows negative relationships. Widths of arrows and adjacent values indicate standardized effect size of each predictor variable. Asterisks denote significance levels: *p < 0.05; **p < 0.01; ***p < 0.001. Gray standardized effect sizes represent relationships of only marginal significance (*p* < 0.1), and gray arrows are nonsignificant (*p* > 0.1); *N* = 48

For the structural equation model (*SEM*; Figure 4a), we investigated all single term relationships of SNHs at the field scale (distance to SNH and SNH type) and at the landscape scale (percentage and diversity of SNHs in 1,000‐m radius around the plots) on wheat yield of experimental plots and the four selected pest rates as mediator between SNHs and wheat yield.

The final *SEM* fitted the data well (best simplified model: C_30_ = 15.33; *p* = 0.99; Figure 4b), and none of the independence claims remained significant, indicating that no important links were missing in the model. In the final model, wheat yield of experimental plots was no longer affected by distance to or type of SNH (Table 4). Weed cover and percentage of SNHs were the only variables having direct, marginal negative effects on yield of experimental plots (standardized effect size for weeds: −0.29; *p* < 0.1; for %SNH: −0.40; *p* < 0.1). Weed cover also took a larger share of the indirect effect of distance to a SNH on wheat yield (indirect standardized effect size: 0.153; Table S3) than any other pest group. However, the marginal negative impact of percentage of SNHs on yield could not be explained by the selected pests.

Fungal infection rates were unaffected by single term effects of SNHs, and herbivory rate was only marginally influenced by SNH type. Weed cover diminished significantly with increasing distance to a SNH but was also slightly reduced by the percentage of SNHs in the surrounding. Also, fungal seed and fungal leaf infection, herbivory by CLB larvae, and weed cover remained uncoupled from each other (Figure 4b; Table 4).

## 4 DISCUSSION

In this study, arable weeds could be identified as the only putative culprit (albeit only on a 10% significance level) out of the four selected pests causing yield losses close to semi‐natural habitats (SNHs). Weeds had a significant negative effect on yield only when we confined our analysis to the two proximate distances to a SNH (yield at 1‐m and 5‐m distance). Our structural equation model (*SEM*) confirmed that wheat yield tended to be affected only by weed cover representing the most important mediator between distance to a SNH and yield of the investigated pests in this study. In addition, the *SEM* revealed a marginal negative influence of percentage of SNHs in the landscape on wheat yield of the experimental plots.

### 4.1 Weed cover as culprit for yield losses at SNHs

Weed plants are competitors for light, nutrients, and water to the crop plant, and most weed species are adapted to the agricultural habitat (Gallandt & Weiner, 2015). However, in this agricultural management system, weed plants cannot establish permanently within the fields due to intensive and regular use of herbicides and tillage (Geiger et al., 2010; Isenring, 2010). Therefore, SNHs may represent refugia and source habitats for weeds and enable a constant recolonization of arable fields (Baudry et al., 2000; Lozada‐Gobilard et al., 2019). Distance restrictions of pesticide applications to SNHs set by EU regulation (No. 1107/2009) and detailed by the German plant protection law (Deutscher Bundestag, 2012) prohibit to apply herbicides in direct proximity to a SNH. Hence, the result that weed cover was especially affecting yield at the most proximate distances to a SNH can be explained by the interplay of SNHs as source habitat for weeds and regulations to pesticide applications: While weed cover was still elevated close to the SNH, herbicides may be the cause of rapid decrease toward the field centers. This pattern is exactly opposite to the effect of distance on yield: With increasing distance to a SNH, yields rapidly recovered to mid‐field yields. Consequently, weed cover seemed to be the most promising biotic candidate of the investigated pests to explain parts of yield losses at the field scale, especially as the indirect effect of distance to a SNH is foremost taken up by weed cover compared with other pests. Considering, however, the larger direct effect of distance to a SNH on yield than the effect mediated through weeds (Table S3), wheat yield close to SNHs might rather be restricted by abiotic conditions, such as shading by the SNH itself as elaborated in Raatz et al. (2019).

### 4.2 The potential of fungal infection and herbivory of CLB due to an adjacent SNH

Remarkably, yield was unaffected by fungal seed and fungal leaf infection rate as well as herbivory rate of CLB larvae. Thus, we have to assume that the unspecific fungal rates and the herbivory of the selected animal pests were not the causes of yield reduction close to a SNH. Yet, fungal leaf infection and herbivory of CLB were affected in different ways by the type and distance to a SNH whereas fungal seed infection remained unaffected of SNHs.

The latter result is rather unexpected because several studies have shown that seed‐inhabiting fungi can be associated with a variety of noncrop plants. Especially for phytopathogenic fungi of the genus *Fusarium*, Fulcher et al. (2019), Fulcher et al. (2020)) and Suproniene et al. (2019) observed a remarkable influence of weed patches to an increased incidence of *Fusarium graminearum*, the causal agent of Fusarium head blight, on wheat plants. The authors found a high *Fusarium* abundance on several noncrop grasses providing a permanent habitat for the fungi, especially in the overwintering periods. We also expected these relationships between the grass verges of our investigated kettle holes and hedgerows and therewith hypothesized an increased fungal seed infection rate in the proximity of the SNHs. However, our study did not confirm this pattern for the year of investigation. One explanation could be that the incidence of *Fusarium* on noncrop grasses and the dispersal to the wheat plants is strongly affected by annual and regional environmental conditions, mainly by precipitation, humidity, and weed density (Fulcher et al., 2020). We assume that the relatively dry year 2016 influenced the abundance of the total fungal infection rates (here: median 5.6%) as well as the fungal population structures on wheat plants. The high proportion of *Alternaria* fungi in the total fungal seed infection rates indicates this influence of low air humidity: This genus develops and spreads very homogeneously in wheat fields under warm and dry environmental conditions (Schiro et al., 2018, 2019). At the same time, *Alternaria* fungi can act as a competitor to *Fusarium* fungi in the same habitat and suppresses its growth (Müller et al., 2015), which we might see in this study: 46% of the seed‐inhabiting fungi were *Alternaria* fungi, but only 2% of them were identified as *Fusarium* fungi. A wetter year may completely change this population structure and favor *Fusarium* fungi instead (Müller et al., 2016). A multiyear investigation and a comprehensive analysis of the fungal community are thus needed to better understand the underestimated relationships between fungal seed pests on grassy weeds and their impact on crop production and yield losses.

Although fungal leaf pathogens had no significant effect on yield, they might have been promoted by an adjacent SNH as those harbor alternative host plants. We could show that infection rates were elevated at wheat plants close to kettle holes compared with those in field interior. However, we could not confirm this trend at hedgerows. As humidity is of particular importance for fungal infections (Fig ueroa et al., 2018; Savary et al., 2015), the grass verge around kettle holes might have provided a more suitable habitat than the vegetation in hedgerows and therewith fungal populations could spillover from kettle holes more effectively.

Herbivory rates of cereal leaf beetles (CLB) had also no effect on wheat yield. This might be due to the fact that herbivory was predominantly affected by SNH type, which in turn had no effect on yields of experimental plots. Herbivory was on average three times higher on plots adjacent to kettle holes compared with those at hedgerows. This stands in contrast to the fact that woody habitats account for more than a third of the explained variance of adult densities of CLBs in agricultural landscapes (Sawyer & Haynes, 1986), as sexually immature adults of CLBs overwinter predominantly in woody habitats under bark or leaf litter from which they spillover in spring to colonize cereal fields (Buntin et al., 2004). Nonetheless, Honek (1991) showed that the females of CLB preferably select plants with a higher water content to deposit their eggs. Thus, higher herbivory rates of CLB larvae at wheat plants close to kettle holes could be due to a higher water content of the leaves and therewith a preferred site for hatched CLB larvae.

### 4.3 Inconsistent responses of pests to landscape complexity

Fungal leaf infection rates in proximity of a SNH were enhanced with increasing percentage of SNHs at a landscape scale. Hence, fungal leaf pathogens might have profited by a higher share of alternative host plants within SNHs in the surroundings. This stands in contrast to Papaïx et al. (2014) who demonstrated that a diverse landscape of susceptible and resistant host plants to fungal plant pathogens was found to be more efficient in impeding the distribution of the pathogen.

In contrast, damage rates of the selected animal pest, CLB, were not affected by the percentage of SNHs, but rather by the interplay of SNH type and diversity of SNHs in the landscape: Herbivory increased at kettle holes whereas it decreased at hedgerows with increasing diversity of SNHs within a 1,000‐m radius. Natural enemies, such as ladybirds, lacewings, parasitic wasps, and hoverflies, are known to respond positively to landscape complexity (Chaplin‐Krameret al., 2011; Veres et al., 2013) and are more likely to perform pest control in SNH‐rich surroundings (Grab et al., 2018; Martin et al., 2015). However, natural enemies of CLBs might have been more abundant at hedgerows compared with kettle holes embedded in agricultural fields because woody habitats and other perennial field boundaries represent important habitats for many insect species (Holland & Fahrig, 2000; Morandin et al., 2014). In contrast, we have kettle holes, where zoophagous animals are more likely to be restricted to water surfaces and do not colonize arable fields in such extent (but see Raitif et al., 2019). Hence, in our study area, at kettle holes, landscape complexity might have attracted pest population and therewith herbivory rates of CLB larvae, whereas at hedgerows, landscape complexity might have increased predator populations of CLBs and feeding rates decreased.

These inconsistent responses of landscape complexity depending on SNHs at the field scale on pest groups emphasize that managing pest populations of one crop species might require different landscape properties and should be taken into consideration when designing agriculture landscapes supporting naturally provided ecosystem services.

### 4.4 Negative effects of landscape complexity on yield

We observed a minor reduction in wheat yield in the intensively managed fields with an increasing share of SNHs in the landscape. In terms of landscape structure, a higher share of SNHs was closely linked to a higher edge density (ED) (% SNH and ED in 1,000 m: *r* (46) = 0.86, *p* < 0.001) and smaller fields (% SNH and field size: *r* (46) = −0.44, *p* < 0.01). A higher share of field borders—that restrict the use of pesticides and fertilizers in the studied agricultural system—added to an overall negative effect of SNHs in the landscape.

Deng et al. (2017) postulated that the positive ecological effect of landscape complexity on crop production is ruled out by the strength of the negative effect of reducing cultivated land, so that at the end the net effect of landscape complexity on crop production is slightly negative. Perhaps in our study area, due to intensive agricultural management positive ecological effects of SNHs might not have come to play.

## 5 CONCLUSION

In the present study, arable weeds tended to be the only putative culprit of the biotic factors that might have been responsible for yield reduction in proximity to semi‐natural habitats (SNHs) within conventionally managed wheat fields. However, the negative effect of weeds on yield was only measurable in the proximity of the SNH within 5 m from the field border, where pesticide application is prohibited due to distance regulations. Hence, in our study system, potential spillover effects of the investigated pest groups might have been impeded by farming practices. Unfortunately, information on pest management was not available and we have to point out that yield depends most likely on multiple factors including nutrient availability. Further studies should incorporate a wider set of yield drivers, including biotic and abiotic drivers as well as farming practices.

Our study presents a step toward understanding the role of SNHs for crop production, and it emphasizes that in intensively managed systems spillover from adjacent SNHs—may it be pest or predator populations—can be overshadowed by crop management. Hence, our data may serve as a baseline to compare ecosystem services of SNHs and landscape diversity on agricultural yields in less intensively used agricultural systems.

## CONFLICT OF INTEREST

The authors state no conflict of interest.

## AUTHOR CONTRIBUTIONS

**Larissa Raatz:** Conceptualization (supporting); Data curation (lead); Formal analysis (equal); Investigation (lead); Methodology (equal); Visualization (lead); Writing‐original draft (lead); Writing‐review & editing (equal). **Karin Pirhofer Walzl:** Conceptualization (equal); Funding acquisition (equal); Project administration (equal); Supervision (supporting); Writing‐review & editing (equal). **Marina E. H. Müller:** Investigation (supporting); Methodology (supporting); Resources (equal); Supervision (supporting); Writing‐review & editing (equal). **Christoph Scherber:** Conceptualization (equal); Formal analysis (supporting); Funding acquisition (supporting); Methodology (supporting); Supervision (equal); Validation (equal); Writing‐review & editing (equal). **Jasmin Joshi:** Conceptualization (equal); Funding acquisition (equal); Methodology (supporting); Project administration (equal); Resources (equal); Supervision (equal); Validation (equal); Writing‐review & editing (equal).

## Supporting information

Supplementary Material

## Data Availability

Detailed primary data and the R code are stored and published in the ZALF repository and are available online at https://doi.org/10.4228/zalf.bygf‐e4bb (Raatz et al., 2021).
