# Supplementary material for: Who is the culprit: Is pest infestation responsible for crop yield losses close to semi‐natural habitats?
Source: Ecol Evol. 2021 Aug 24;11(19):13232–46. doi: 10.1002/ece3.8046 (PMC8495789; doi:10.1002/ece3.8046)
Supplement: Supplementary file 1 — Supplementary Material [file ECE3-11-13232-s001.docx]

**Supporting Information to**

**Who is the culprit: Is pest infestation responsible for crop yield losses close to semi-natural habitats?**

Raatz L, Pirhofer Walzl K, Müller MEH, Scherber C, Joshi J

**Table S1**: Scoring scale from 1 (no damage) to 9 (very severe damage), adapted from Moll et al. (2000), indicating the percentage of damaged flag leaves by cereal leaf beetle (CLB) larvae

| **Scoring** | **Percentage values** |
| --- | --- |
| 1 | 0% |
| 2 | > 0 - 2% |
| 3 | > 2 - 5% |
| 4 | > 5 - 9% |
| 5 | > 9 - 14% |
| 6 | > 14 - 20% |
| 7 | > 20 - 35% |
| 8 | > 35 - 60% |
| 9 | > 60 - 100% |

**Table S2**: Conversion of weed cover recorded with Braun-Blanquet (1951) scale to percentage values.

| **Braun-Blanquet scoring** | | **Percentage values** |
| --- | --- | --- |
| 1 | 1 small individual | 0.1% |
| 2 | 2 - 5 small individuals | 1% |
| 3 | 6 - 50 small individuals | 2.5% |
| 4 | > 50 small individuals | 5% |
| 5 | 5 - 15% coverage | 10% |
| 6 | 15 - 25% coverage | 20% |
| 7 | 25 - 50% coverage | 37.5% |
| 8 | 50 - 75% coverage | 62.5% |
| 9 | 75 - 100% coverage | 87.5% |

**Table S3:** Relative strengths of the direct and indirect effects in the final piecewise SEM examining relationships between local (distance to a SNH) and landscape factors (percentage of SNHs) of SNHs, crop pests (fungal leaf infection and weed cover) and winter wheat yield (Fig. 4).

| **Response variable, predictor,**  **and type of effect** | **Mediator variable** | **Standardized path coefficient** |
| --- | --- | --- |
| Winter wheat yield of exp. plots |  |  |
| Distance to a SNH |  |  |
| Direct | none | 0.207 |
| Indirect | Fungal leaf infection | -0.016 |
| Indirect | Weed cover | 0.153 |
| Percentage of SNHs |  |  |
| Direct | none | -0.398 |
| Indirect | Fungal leaf infection | 0.051 |
| Indirect | Weed cover | 0.101 |

*Note:* Fungal seed infection and herbivory of CLB larvae as mediator are not included because both are no longer predictors of yield in the final piecewise SEM (Table 4).

**Figure S1:** Effects of a – c number of fungal colony forming units (CFUs) per 100 wheat seeds, d – f the percentage of fungal leaf infection, g – i the percentage of herbivory (> 10% per leaf) caused by CLB larvae and j – l weed cover on winter wheat yield measured as seed biomass in t ha^-1^ of the experimental plots (N = 48). The three subfigures per pest group present the single term effect on yield, their effect depending on distance to the SNH and their effect depending on type of SNH. Curves represent fitted values according to a linear mixed-effects model 2a. All relationships were non-significant (P > 0.05).

**References:**

Braun-Blanquet, J. (1951) *Pflanzensoziologie: Grundzüge der Vegetationskunde*. 2nd edn. Wien: Springer-Verlag.

Moll, E., Flath, K. and Piepho, D. (2000) *Methodische Anleitung zur Bewertung der partiellen Resistenz von Getreidesortimenten und die SAS-Applikation RESI*, *Testing of Crop Cultivars for Resistance to Noxious Organisms at the Federal Biological Research Centre*. Berlin, Prarey.
